# Simulation based scheduling of crew routing with learning and forgetting effects in repetitive projects

**DOI:** 10.1038/s41598-024-82389-5

**Published:** 2025-02-28

**Authors:** Tayseir Hegazy, Khaled Hamdy, Hatem El-Behairy, Yasmeen A. S. Essawy

**Affiliations:** 1 Department of Structural Engineering, Ain Shams University (ASU), Cairo, Egypt; 2 Department of Construction Engineering, The American University in Cairo (AUC), Cairo, Egypt

**Keywords:** Delay risk assessment, Line-of-balance (LOB) scheduling, Repetitive projects, Construction management, Civil engineering, Mathematics and computing

## Abstract

The Line of Balance (LOB) technique is essential for scheduling repetitive projects, offering benefits over the Critical Path Method (CPM) by ensuring consistent workflow and reducing project duration through the learning rate effect. This effect shortens activity durations when the same crew repeats tasks. Consistent crew assignments for each activity are key to maximizing this benefit, as routing crews between activities can disrupt the learning rate momentum. However, crew routing can be cost-effective by lowering indirect costs. This study explores the balance between maintaining distinct crews for each activity and allowing crew routing. Results indicate that while specific crew assignments optimize duration, they require more crews. In contrast, routing reduces the number of crews but extends project duration. The study proposes a simulation approach to optimize crew numbers and movements. However, the study has limitations, as the simulation model must be customized for each specific project, and crew assignments must comply with the model’s requirements.

## Introduction

Construction projects present a distinct array of tasks fraught with numerous challenges and obstacles. Within conventional construction endeavors, these tasks may recur to varying extents. When activities repeat frequently and with remarkable consistency within a project, it qualifies as a repetitive activities project^1^. Such projects demand specialized handling to optimize project duration and activity productivity efficiently. Proper management unlocks the advantages inherent in repetitive projects, provided interruptions are minimized. To achieve uninterrupted scheduling for these projects, numerous techniques have been devised. While conventional network-based methods like the Critical Path Method (CPM) excel in scheduling non-repetitive projects, they falter in handling repetitive activities due to disparities in production rates. This can lead to work stoppages and inefficient resource allocation which eventually will lead to excessive costs when scheduling using CPM method. Also, it is very difficult to represent and visualize the relationships between repetitive activities using CPM. Moreover, the CPM techniques do not deal with two different objectives, which are meeting the desired project deadline, and maintaining work continuity^2^.

Line of Balance (LOB) is a scheduling technique used in project management, particularly in construction projects, to manage and control repetitive processes efficiently. It is especially useful for projects with a continuous and repetitive workflow, such as the construction of high-rise buildings, roads, or large infrastructure projects. The Line of Balance method helps in optimizing resources, reducing costs, and improving project delivery times. LOB represents a variation of linear scheduling methods designed to achieve a balance in operations, ensuring the continuous execution of each activity. The primary advantage of the LOB methodology lies in its ability to present production rates and durations in a readily understandable graphical format. The LOB chart offers a quick overview of the status of an activity’s progress, enabling the identification of existing issues and the anticipation of potential future bottlenecks. Clearly, LOB provides a more insightful understanding of projects involving repetitive activities compared to other scheduling techniques, as it allows for the adjustment of activity production rates. This approach facilitates a smooth and efficient resource flow and demands less time and effort for production compared to network schedules. Dealing with projects with a repetitive nature necessitates the integration of multiple factors that coexist with these projects. One of the most important factors is the learning rate gained due to the project lifespan.

## Background

**Fig. 1:**
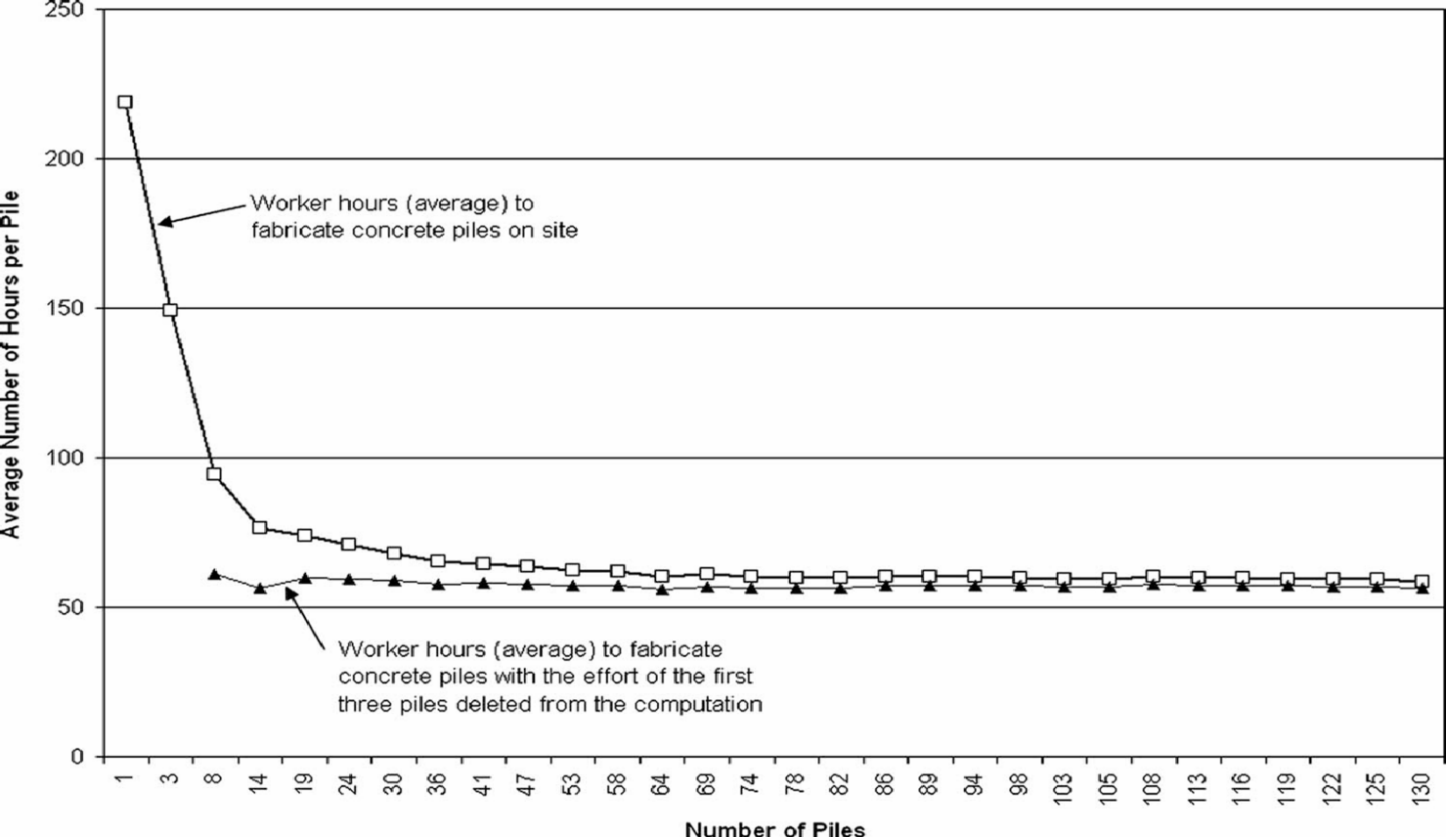
Average number of piles per hour^3^.

One of the most important phenomena related to repetitive projects is the effect of the learning curve. It’s known that operations with a repetitive nature offer higher production when performed repetitively using the same person without interruptions. Hence, labor productivity is enhanced when repetition exists in the project, for example Fig. 1 illustrates the impact of excluding the fabrication times for the first three piles on the cumulative average times for pile fabrication. The analysis shows that while excluding these initial times results in a slightly lower cumulative average.

**Fig. 2:**
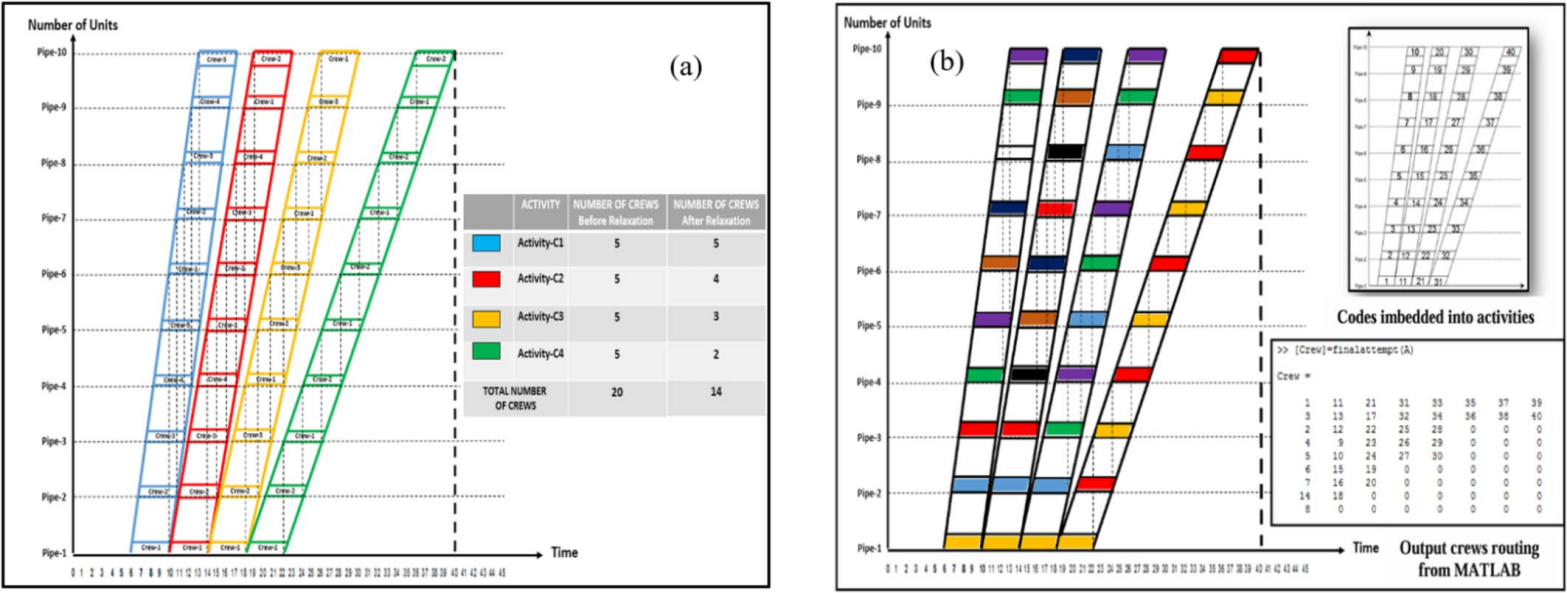
**a** The typical LOB before crew routing and **b** The LOB after implementing crew routing among activities^5^.

Accordingly, the abovementioned phenomena of learning curve, care must be taken when dealing with LOB calculations to keep track of the learning curve’s effect on the duration and production rate of activities. The basic assumption of LOB states that the relationship between time and the number of produced units is linear and constant production rate. This is not true according to the learning curve phenomenon^4^. As stated, the theoretical concept of LOB is to maintain the workflow and prevent interruptions. To achieve that, each crew must work on the same activity till the end of the last unit of the activity. This concept of maintaining the exact same crews for each activity could lead to several disadvantages such as a higher indirect cost of hiring and firing crews, hence, we need a proper resource allocation to avoid this issue. Several methods were developed to re-allocate the crews among activities to minimize the total number of crews needed to fulfill the job. Figure 2 shows an example of a four sequential activities project with 10 units. This project was scheduled using two different scenarios, the first one is to maintain the same crews for each activity with no crew routing allowed, and the second scenario is to re-allocate the crews among activities to reduce to total number of crews required. According to this new allocation of crews, the total number of crews needed to complete the job is only 9 crews in comparison with 14 in the first scenario. This means less hiring and firing of crews will be made. Hence, less indirect cost.

## Literature review

Investigations into Line-of-Balance (LOB) within the construction management literature predominantly center around three key aspects: (1) the equitable allocation of resources, (2) addressing challenges related to meeting deadlines, and (3) analyzing the impact of the learning curve on project duration. In this study the third aspect will be discussed in addition to taking the forgetting rate effect and how to quantify the amount of learning lost during an interruption caused by changing the crew which was not adequately addressed in previous research. For example^5^, . proposed a methodology to optimize the crew routing map among the activities in a repetitive project to reduce the required number of crews needed to finish the project within the required deadline. As observed in Fig. 2, the initial case on part (a) of Fig. 2 required 14 crews to maintain specific crew allocations for each activity, whereas implementing crew routing on the right-side part (b) reduced the number of crews to just 9 crews. The study proves beneficial in reducing the indirect cost of firing and hiring crews by reallocating the crews among activities. But the effect of learning rate was not discussed in that study as it possesses an essential role in project duration and cost results. Changing the crew`s task will lead to a loss of learning gained while repeating a specific activity. Hence, it influences project duration as a result of the learning loss incurred. This loss of learning will lead to an additional cost of increasing the project duration in comparison with the typical LOB crew orientation of maintaining a specific allocation without routing. Therefore, it is essential to develop a new methodology that incorporates both learning and forgetting rates when proposing a crew mapping strategy for routing crews between activities. And carry out a tradeoff between the two methodologies by taking both learning and forgetting effects into account while carrying out the LOB calculations. Quantifying the loss of learning proves valuable for more accurate calculations of project performance, ultimately influencing the project’s duration and cost. Determining project duration becomes complex when considering learning and forgetting rates alongside crew routing among activities using conventional scheduling methods. Therefore, the utilization of simulation software becomes essential for addressing this challenge study has a dual focus. Firstly, it aims to devise a method for quantifying the loss of learning stemming from crew routing during the project’s life cycle. Secondly, is to carry out a trade-off between both methodologies for a better decision-making process.

In their study^6^ presented a comprehensive multi-objective scheduling optimization model tailored for repetitive construction projects, aiming to achieve optimal resource allocation that minimizes both project duration and cost. The model was developed through three key modules—scheduling, cost, and optimization—that work in concert to address the unique challenges of managing concurrent crews, maintaining work continuity, and navigating complex precedence relationships.

The scheduling module harmonizes multiple crews working concurrently on each activity by ensuring work continuity and accounting for variations in productivity rates. The cost module enhances planning flexibility by incorporating various contractual cost components, such as direct and indirect costs, incentives, penalties, and occupation rental costs, providing a realistic view of financial impacts under different contract terms. Finally, the optimization module utilizes a genetic algorithm to determine the optimal number and sequence of crews, identifying resource utilization plans that minimize both time and cost. The model’s effectiveness was validated through an application example from the literature, which demonstrated an 8% reduction in project duration and a 0.78% reduction in total cost compared to existing models. Additionally, a real-world highway development and renovation case study showcased the model’s practical benefits, underscoring its effectiveness in managing large-scale repetitive construction projects.

The study’s primary contributions lie in three key advancements: Concurrent Crew Coordination: The model addresses the challenge of coordinating multiple crews with varying productivity rates while respecting work continuity and precedence constraints. This approach supports efficient management of both standard and unique repetitive activities.

Flexible Cost Representation: By incorporating a wide range of contractual cost options, including incentives, penalties, and rental costs, the model offers planners a realistic representation of financial considerations, allowing them to select the most advantageous cost structure for the project. Diverse Optimal Resource Plans: The model provides planners with a variety of optimal or near-optimal resource utilization plans, enabling them to strike a balance between project efficiency and financial feasibility. This flexibility is particularly valuable for repetitive construction projects, where optimizing time and cost is essential. Despite its contributions, the study has certain limitations. Firstly, the model enforces strict crew work continuity, disallowing interruptions; introducing selective interruptions could further reduce project duration. Secondly, the model does not account for risks and uncertainties related to site conditions, weather, labor productivity, or equipment availability, which can affect construction timelines. Future research will address these limitations, incorporating flexibility for interruptions and mechanisms to account for real-world uncertainties, further enhancing the model’s robustness and applicability in diverse construction scenarios.

## Problem statement

No clear research has been conducted on the trade-off between maintaining specific crews for each task in repetitive projects and allowing crew routing among activities, particularly when considering learning and forgetting rates. This is largely due to the challenge of quantifying the impact of learning and forgetting on activity duration in large-scale projects. Assigning specific crews to each task improves project duration by enhancing the learning rate, but it demands the maximum number of crews, corresponding to the number of activities. Conversely, permitting crew routing reduces the number of crews required but extends the project duration due to the loss of accumulated learning, as forgetting occurs when crews transition between different activities. Addressing this gap would enable more informed decision-making in optimizing crew routing scenarios that align with project objectives.

## Proposed methodology

A case study of a linear construction project with repetitive activities will be tackled (Table 1). First it will be solved using the typical LOB method as the base case to calculate the total duration needed to complete the project using a separate crew for each activity. Then the same case study will be solved again using simulation software incorporating the learning rates to calculate the actual project duration when the learning rate is considered, and continuous workflow is maintained, and no crew routing will be allowed during this trial. A third scenario will be implemented with applying crew routing among activities to minimize the needed resources, hence minimizing the cost. A comparison will be made between all trials’ outcomes of the project regarding the project`s final duration and total cost.

**Table 1:** Scenarios parameters and methodology.

Scenario #	Description	Parameters	Methodology
1	Base Case		LOB using Excel
2	Incorporating Learning	Learning rate	Anylogic
3	Incorporating crew routing with learning and forgetting	Learning rate Forgetting rate Crew alternatives	Anylogic

## Learning rate calculation method

Numerous models have been created to assess the impact of the learning rate on the duration of repetitive activities. Among these models, the log-linear model, commonly known as “Wright’s model,” is frequently employed. Its primary application involves determining the duration of an activity when repeated a specific number of times.

1 $$\:{y}_{x}=\:{C}_{1}{X}^{-b}$$

In this equation, “y” represents the average time required to produce a unit number “x”. The variable “C1” corresponds to the time required for manufacturing the first unit, and “b” represents the learning index coefficient. The learning index coefficient “b” spans a range from − 1 to 0, where values closer to -1 indicate a higher learning rate compared to values closer to 0^2^.

## Forgetting rate calculation method

The theory that forgetting curves stem from exponentially decaying memory traces prompts the question of how these traces combine to match empirical data accurately extensively examined this issue, discovering that behavioral power-function forgetting curves arise when the half-time constants of these exponential functions follow a power-function distribution. The parameters of this distribution directly influence the observed rate of forgetting. Furthermore^7^, applied a forgetting model within a Hopfield neural network, successfully replicating empirical power-function forgetting curves. This evidence underscores that power functions effectively describe forgetting curves. In this section, we categorize various forgetting models proposed in the literature into six general types:

- Models based on the learn-forget-learn (LFL) model by^7^.
- Models based on the learn-forget-curve model (LFCM) by^8^.
- The variant regression to invariant forgetting (VRIF) model by^9^.
- The recency model by^10^.
- The power integration diffusion (PID) model by^6^.
- Miscellaneous modeling approaches.

These general models are presented using unified notation and evaluated for their suitability in the context of mixed-model line scheduling. It’s important to note that while most of these formulations were initially developed for lot sizing or worker assignment problems rather than scheduling, the parameters and variables are adjusted accordingly in this discussion.

### LFL-based models

One of the most common models is the learn-forget-learn (LFL) model introduced by^8^. This model calculates the effect on production and the impact of interruptions on the calculated results if they occur.

2 $$\:{p}_{r}=\text{max}\left({p}_{1}\cdot\:{n}_{r}^{-LR},P\text{m}\text{i}\text{n}\right)$$

Where, $\:{p}_{1}$ is the normal processing time for the first produced unit, representing the original activity duration without any learning reduction. The level of experience gained, or learning, is represented by the number of units produced without interruptions *n*_*r*_ and LR is the given learning rate. This formula calculates the new activity duration after learning occurs, which changes with each unit due to the increasing number of units produced without interruptions *n*_*r*_. The reduction of time calculated from the equation cannot exceed the most achievable reduction that could be reached, and a minimum processing time is limited to Pmin as a result of assuming around 80% of the production nature of the activity^11^. To account for the forgetting effect, the following formula was developed^8^.

3 $${{\text{n}}_{\text{r}}}{\text{= }}{{\text{n}}_{{\text{r}} - {\text{1}}}}^{{{\text{1}}+{\text{VR}}/{\text{LR}}}}.{\text{ }}{\left( {{\text{n}}{{\text{r}}_{ - {\text{1}}}}+{\text{ }}{{\text{w}}_{\text{r}}}} \right)^{ - {\text{VR}}/{\text{LR}}}}+{\text{ 1}}$$

After an interruption, the effective volume, denoted as n_r_, needs to be adjusted to account for the impact of forgetting. This adjustment considers a constant forgetting rate, VR, which lies in the range between 0 and 1, as discussed by Jaber et al. (2003). Visually, this adjustment results in a backward shift along the learning curve. In learning curve models, experience is quantified using the learning-relevant volume, nr, at a specific position, denoted as r, in the schedule. Equation (2) demonstrates that n_r_ is a function of the effective volume before the interruption, nr-1, and the number of jobs, w_r_, that could have been produced if the interruption had not occurred. Empirical studies, including^12^, have shown that the extent of forgetting depends on both the experience gained before the interruption and the duration of the interruption, which is used to derive w_r_. If w_r_ = 0 and no interruption of significant duration has occurred, the right-hand side of the equation simplifies to n_*r*−1_ + 1. However, if an interruption takes place between sequence positions r-1 and r, the volume nr-1 will be reduced accordingly^13^.

## Model development environment

Simulation software is vital in construction engineering for planning, analyzing, and optimizing projects. It enables professionals to simulate scenarios, assess risks, and enhance efficiency. Among these tools, AnyLogic is notable for its versatility. Simulation software models and analyzes system behavior over time, helping users understand interactions and the impact of variable changes on performance. Thus, it will be employed in this study to simulate the proposed crew mapping and accurately calculate results, which would be highly complex without the use of simulation software.

## Model development

This research examines a real-life pipeline construction project involving 30 segments, each with its own set of activities, durations, and predecessors. Detailed information, including the specific durations of each activity and the logical sequence of their predecessors, is provided Table 2. As highlighted in the table, all activities are assigned baseline durations and their respective predecessor relationships. The primary assumption underlying the project schedule is that each activity is performed by a single crew, without any additional resources. This assumption serves as a foundation for further analysis of productivity, learning, and crew efficiency throughout the project’s timeline. And it will be the base case for the analysis.

**Table 2:** Project activities with durations and predecessor.

Activity Name	Activity ID	Predecessor	Duration (Days)
Maintenance of Equipment	A	-	100
Locating and clearing	B	-	200
Transport of Aggregates	C	-	150
Transport of Pipes	D	A - B	300
Excavating	E	B	400
String Pipes	F	D	300
Lay Aggregate	G	E - C	100
Lay Pipes	H	G - F	200
Testing	I	H	300
Backfilling	J	I	100
Compacting	K	J	200

### Scenario1: base case calculations

In the initial scenario, the project duration was calculated using traditional Line of Balance (LOB) methods in Excel. The results, presented in Table 3. were computed without accounting for the effects of the learning rate on activity duration, and no crew routing nor work stoppages occurred during this phase. This scenario yielded a total project duration of 21,800 h. The primary advantage of this method is maintaining workflow and preventing interruptions. This results in a higher learning rate, leading to shorter project durations due to decreased activity durations. To calculate the project duration with the learning rate factored in, scenario 2 was conducted.

**Table 3:** Activities start and end dates based on typical LOB calculations.

Activity	Duration (hrs.)	Total Duration	Unit 1 start	Unit 1 end	Unit 30 start	Unit 30 end
A	100	2,900	0	100	2,900	3,000
B	200	5,800	0	200	5,800	6,000
C	150	4,350	0	150	4,350	4,500
D	300	8700	200	500	8,900	9,200
E	400	11,600	200	600	11,800	12,200
F	300	8,700	500	800	9,200	9,500
G	100	2,900	9,300	9,400	12,200	12,300
H	200	5,800	9,400	9,600	15,200	15,400
I	300	8,700	9,600	9,900	18,300	18,600
J	100	2,900	15,700	15,800	18,600	18,700
K	200	5,800	15,800	16,000	21,600	21,800

### Scenario #2: incorporating learning rate

**Fig. 3:**
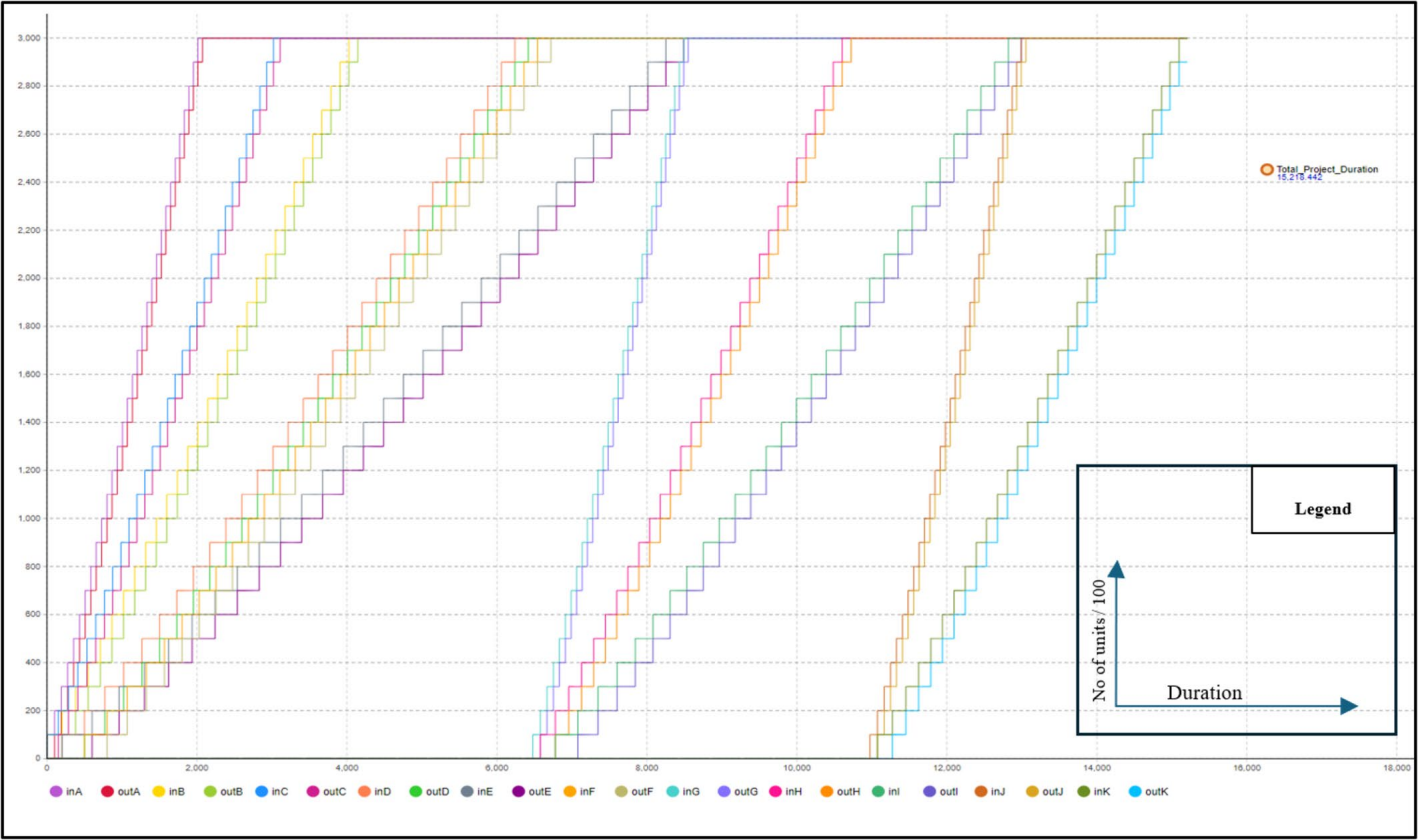
Data extracted from any logic model of scenario #2 lob.

**Fig. 4:**
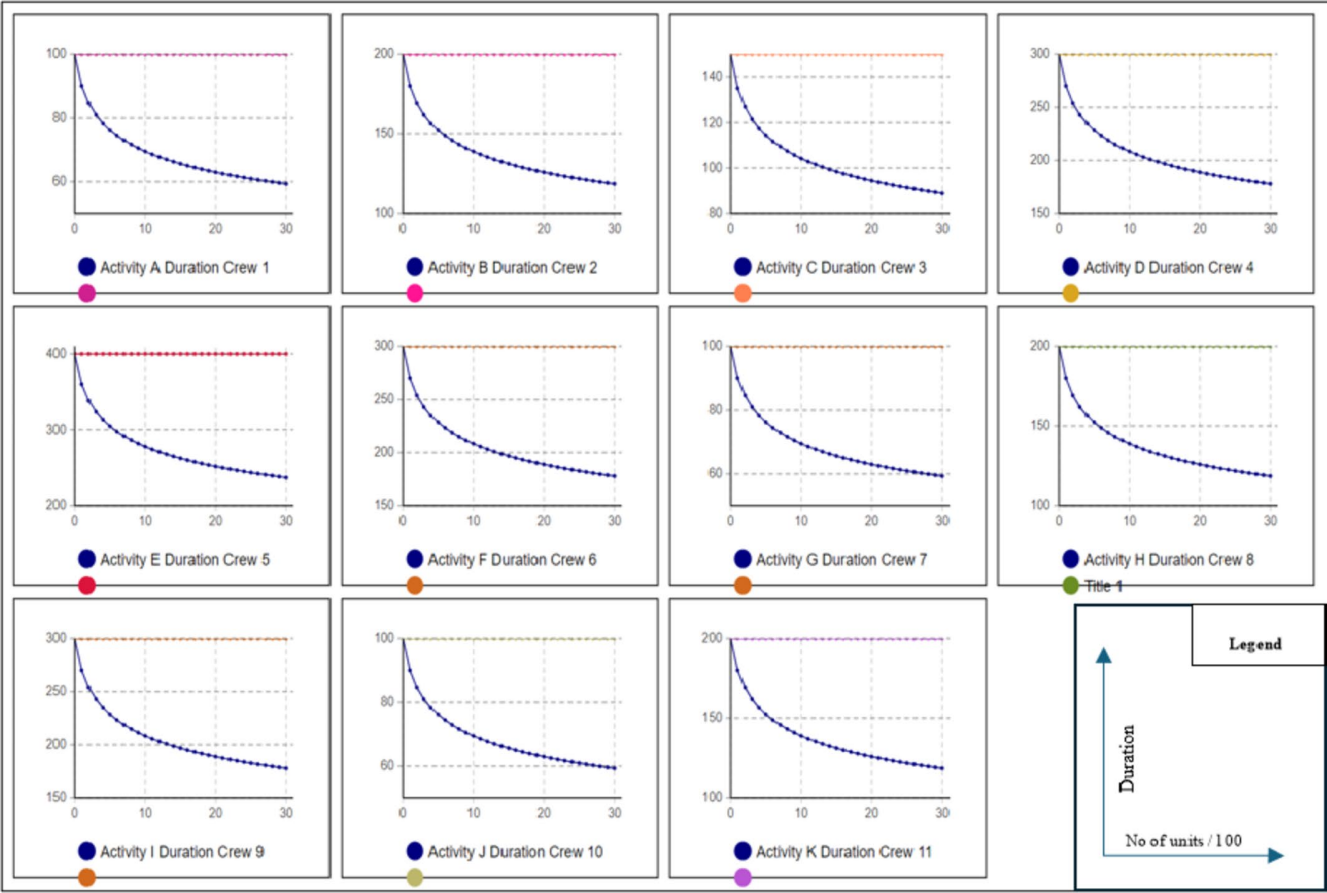
Data extracted from any logic model of the exponential decrease of activity duration as a result the learning rate scenario #2.

As previously mentioned, the primary advantage of scenario #1 is maintaining workflow and preventing interruptions. This results in a higher learning rate, leading to shorter project durations due to decreased activity durations. To calculate the project duration with the learning rate factored in, scenario 2 was conducted. In this trial, a user-defined learning rate of 80% was used to calculate the reduction in activity duration for repeated tasks. Equation 1 was applied to determine the duration reduction. AnyLogic is used to calculate the new project duration, accounting for the learning effect. This trial resulted in a total project duration of 15,218 h with a reduction of 6,582 in comparison with scenario #1 as a result of incorporating the learning rate into calculations. Figure 3 represents the line of balance diagram of this Scenario extracted from AnyLogic. To visualize the impact of the learning rate on activity duration for repeated tasks, activity duration graphs were generated from the model. These graphs illustrate the decrease in activity duration when the same crew performs the task repeatedly without interruptions or crew changes Fig. 4. In third scenario, crew routing will be allowed among activities to reduce the total amount of resources needed.

### Scenario #3: incorporating crew routing with learning and forgetting rates

In this scenario, crews will be alternated and routed among various activities. This strategy aims to reduce the number of crews needed to complete the project, thereby minimizing the costs associated with hiring and firing personnel. However, while this approach helps lower indirect costs, it also increases the overall project duration, in comparison with Scenario #2. The increase in duration results from the disruption of the learning rate momentum when crews change tasks. The following section details how the model interacts to calculate these adjusted durations.

#### Model description

The model follows a sequential process where it initiates with the first unit of an activity if its predecessor is completed. If no work stoppage occurs, the model progresses to the next unit, incrementing the accumulated number of produced units (Nr) represented as X in the Wright model equation Eq. 1. This in turn, impacts the activity duration by decreasing it due to the learning rate equation. If a work stoppage occurs, a parallel model is instantly activated to calculate the number of units that could have been produced if the work stoppage hadn’t happened (Wr). This parameter influences the activity duration by modifying (decreasing) the Nr as a result of the LFL forgetting rate model. The model repeats this process before commencing each activity unit. This stoppage occurs for one of two reasons. The first reason is that the predecessor activity is not yet completed. However, this situation is avoided by incorporating user-inputted lags into the model to ensure continuity of work. The second reason, which will occur in the model, is when the crew is reassigned to a different activity. In this case, the crew moves to the new activity, and the parallel model begins calculating the forgetting effect on the activity the crew just left. The crew will then start building learning momentum in the new activity until they are reassigned again, and this cycle continues.

**Fig. 5:**
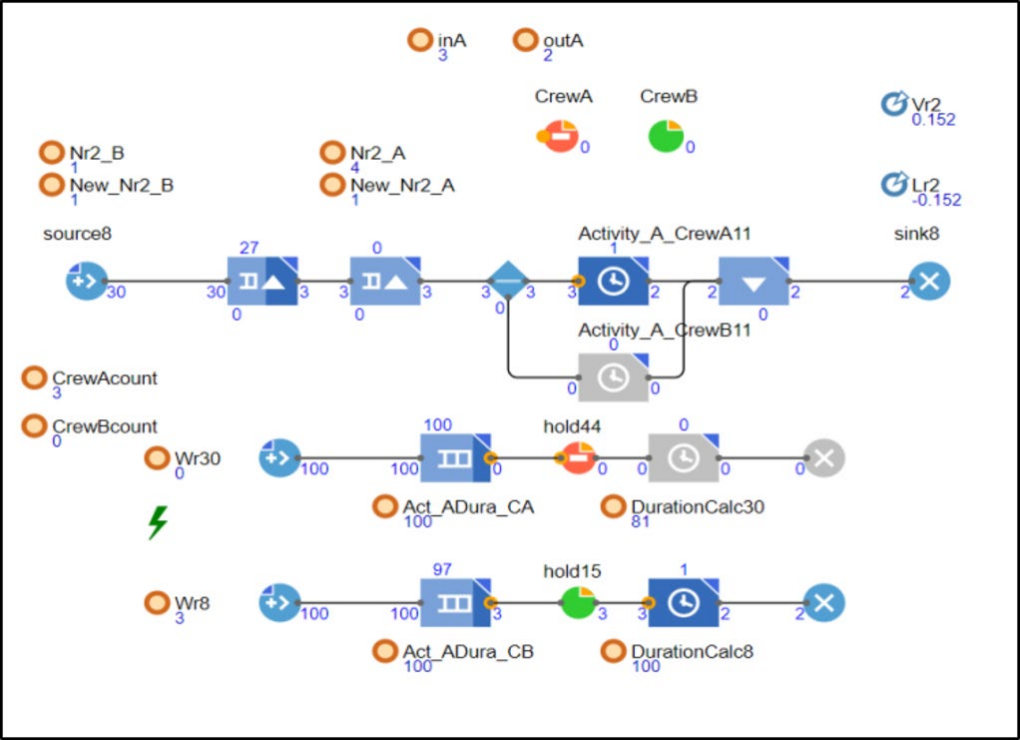
Snapshot from the model demonstrates activity A advancing without any work interruptions.

The chart depicted in Fig. 5 illustrates a simulated execution of activity A, initially scheduled for 100 h, with a learning rate set at 0.152 (80%). As observed in the Fig, the primary model successfully completed 2 units of the activity and is currently processing unit number 3. Notably, there was no work stoppage encountered for crew 1 at this stage. This scenario yielded an accumulated total of uninterrupted units, denoted as Nr, amounting to 4 units. The activity duration was notably reduced to 81 h, influenced by the data computed by the write model underlying the model that was incorporated into the simulation. If the process continues with the same momentum without interruptions, the time required for producing 30 units is 2,072. In comparison to 3000 h required for producing unit number 30 in the base case calculations (Table 4). If we apply the Wright model in Excel or calculate it manually, we will get identical results: 81 h are needed to produce unit number 4, and a total of 2,072 h are required to produce the first 30 units.

**Fig. 6:**
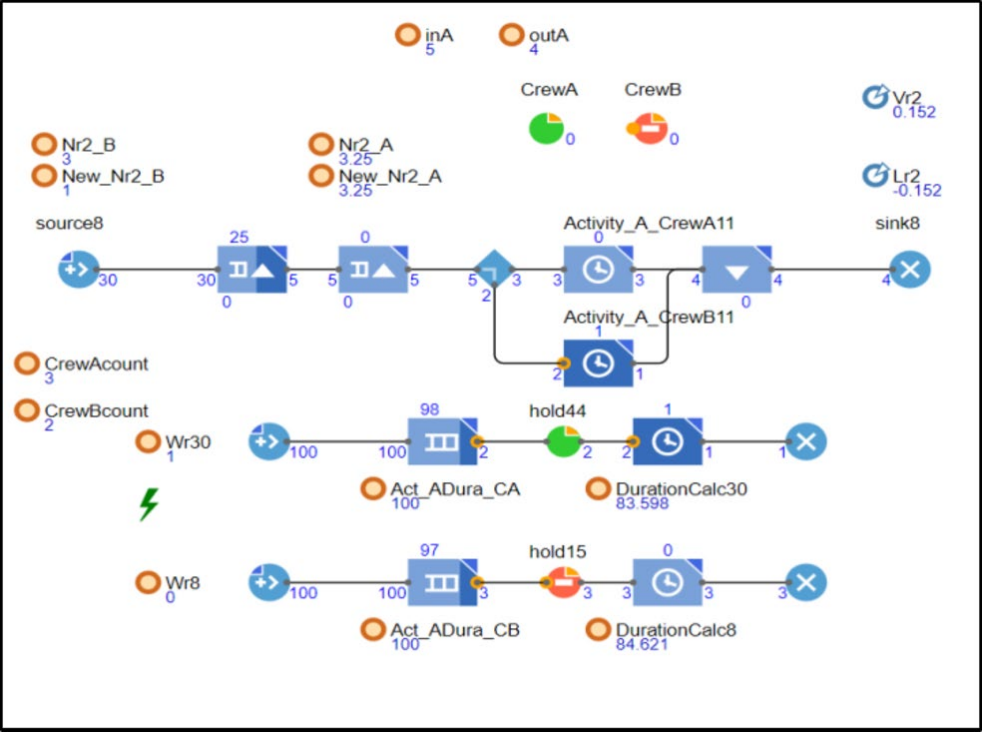
snapshot from the model demonstrates activity A advancing with work interruptions.

As the model progresses, the crew for activity A has been switched to the alternative crew listed in Table 3, from Crew 1 to Crew 4. As shown in Fig. 6, this new crew has successfully completed one unit and is currently working on the second unit. This change has caused a lapse in Crew 1’s learned duration. Since Crew 1 is no longer working on the activity, the parallel model is calculating how many units could have been produced if Crew 1 had continued, thus modifying the accumulated number of units (Nr) and reducing the activity duration. Consequently, the activity duration has decreased from 81 to 83.598 and will continue to decrease as long as Crew 1 remains inactive in this activity. Meanwhile, the new crew (Crew 4) is gaining learning momentum until they also stop working on the activity.

The model will continue to operate similarly for all activities, using the crew alternatives provided by the user, to calculate the total project duration when using only 4 crews compared to 11 crews in scenario #2. In this trial, both learning and forgetting rates are considered in the calculations.

#### Crew allocation

Each activity can be completed using one of two types of crews, based on a user-defined table that considers the nature of the activity and the crews’ specialty and experience. Table 4 outlines the user-defined crew options for each activity. These alternatives will be utilized in the calculations when completing the project with a limited number of crews. The number represents the crew code.

**Table 4:** Crews’ assumptions for each scenario.

Activity Name	Activity ID	Scenario #1 & 2 Crews	Scenario #3 Crews
Maintenance of Equipment	A	1	1 or 4
Locating and clearing	B	2	3 or 4
Transport of Aggregates	C	3	2 or 3
Transport of Pipes	D	4	3
Excavating	E	5	1 or 3
String Pipes	F	6	2 or 3
Lay Aggregate	G	7	1 or 4
Lay Pipes	H	8	3 or 1
Testing	I	9	4
Backfilling	J	10	3
Compacting	K	11	1 or 4

#### Crew routing procedure

**Fig. 7:**
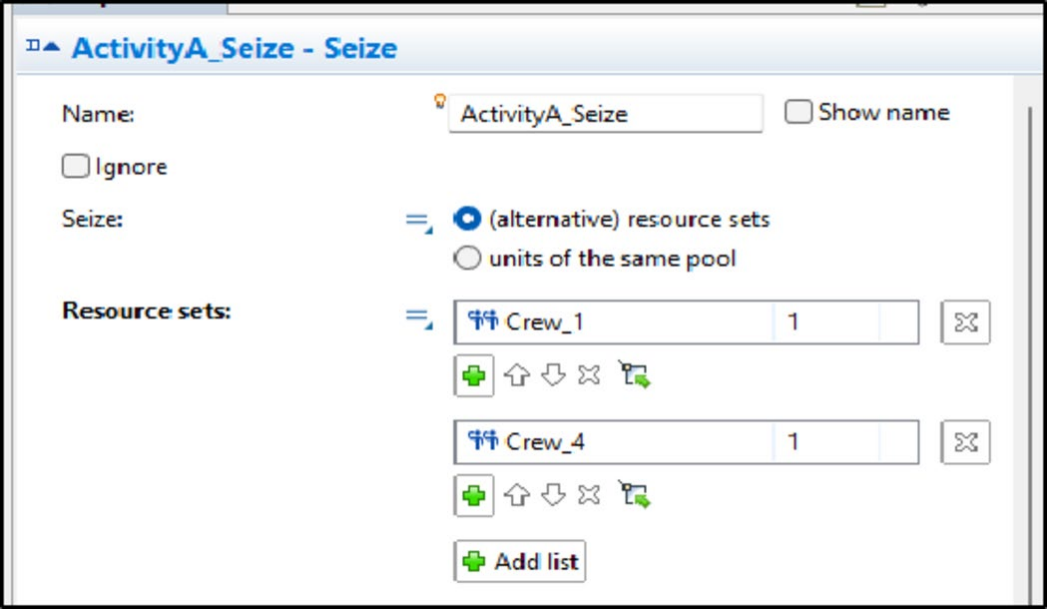
Crew alternatives for activity A in the seize block.

In the model, the crew routing process operates as follows: crew alternatives and options listed in Table 4 are entered into the model within a “size” block. This block is responsible for seizing the necessary resources for each task. It can handle multiple resource options for a single task, seizing the first available option from those provided. For instance, Fig. 7 illustrates the alternatives for Activity A, including Crew 1 and Crew 4. When a unit is ready for processing, the software checks for the first available resource among these options and seizes it. If both options are available simultaneously, a priority can be set by the user for a specific resource, or a random seed can be applied to vary the selection each time, allowing for different options to be tested in each run.

#### Scenario #3 results

**Fig. 8:**
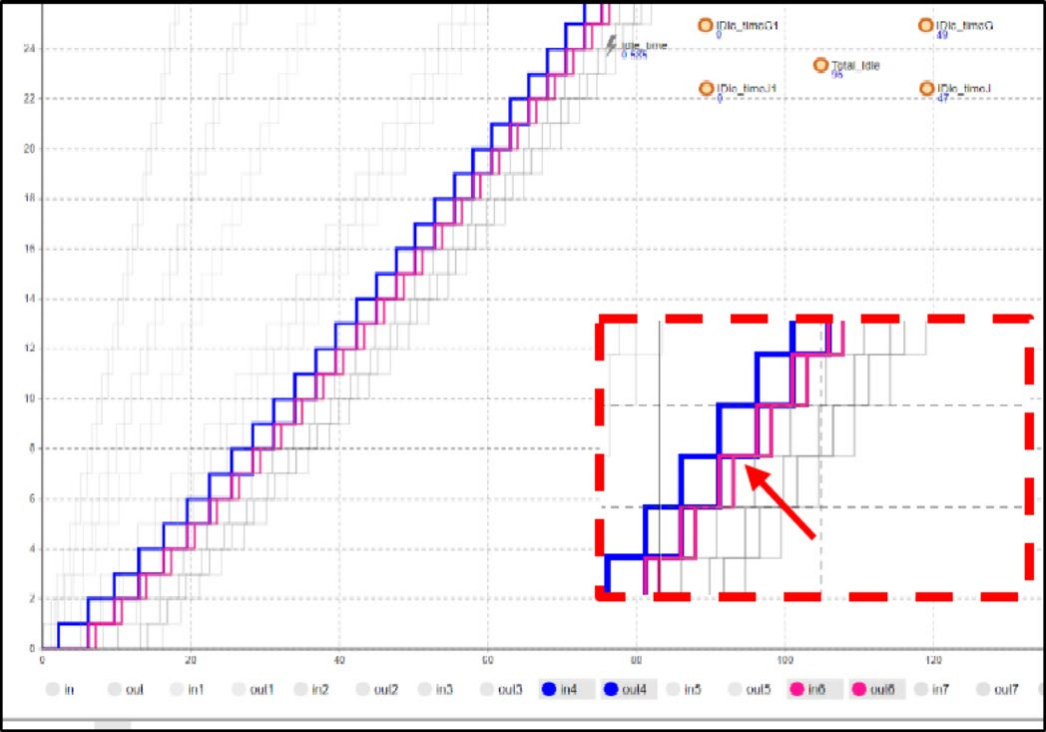
Idle times of activity G that represents the stoppage the activity need to encounter before starting the next unit.

**Fig. 9:**
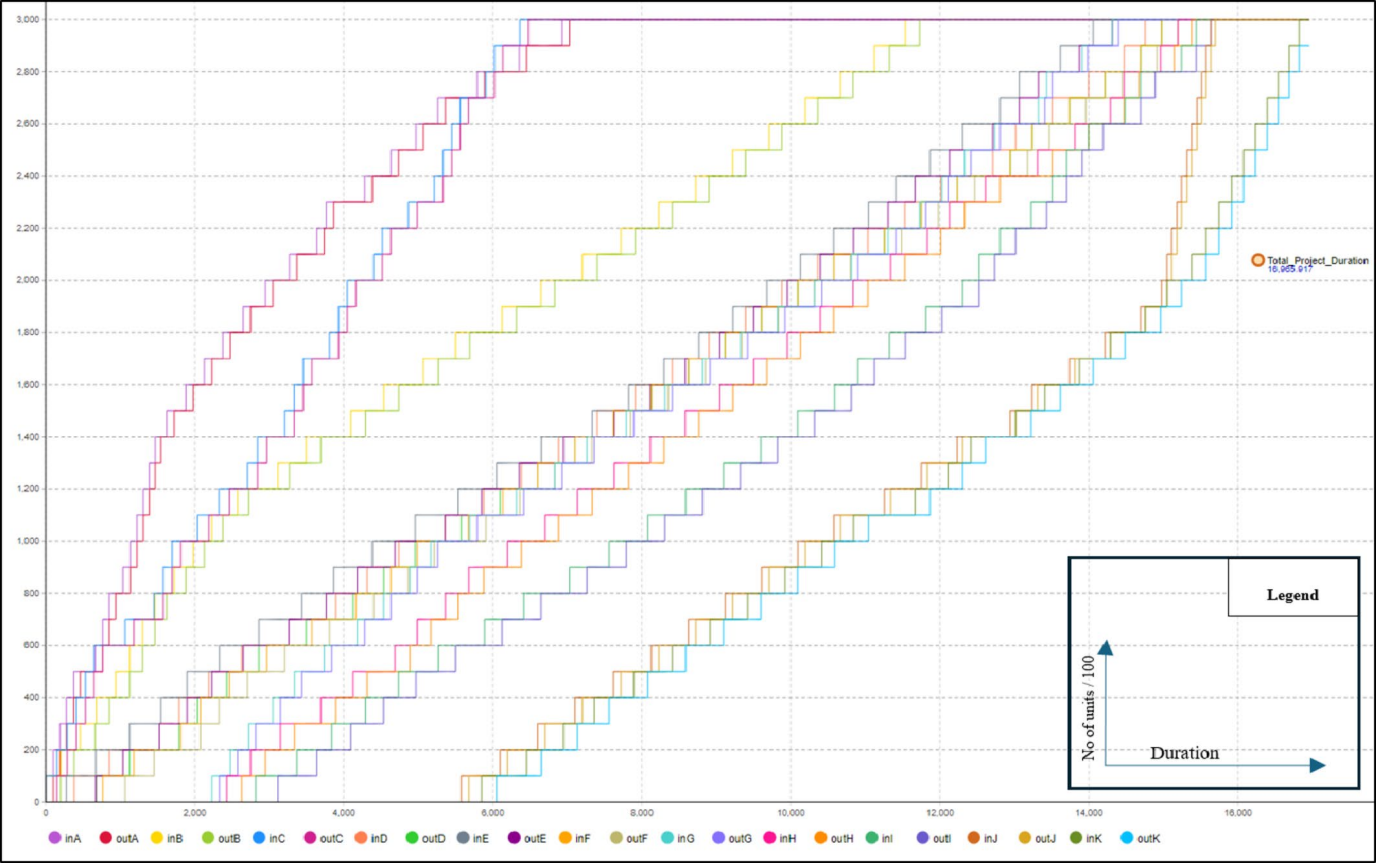
Data extracted from any logic model of scenario #3 LOB.

**Fig. 10:**
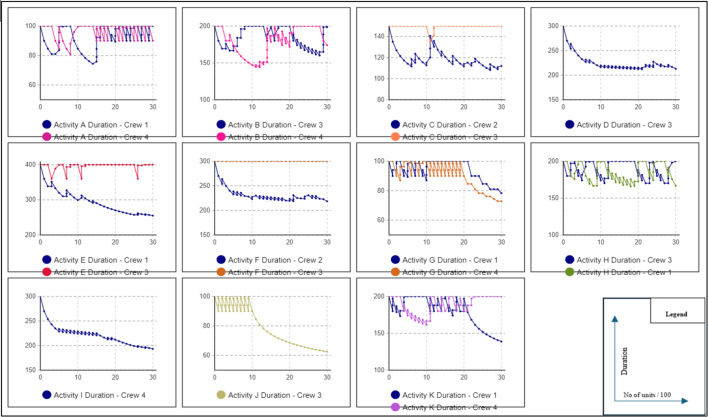
Data extracted from any logic model of the exponential decrease of activity duration as a result of the learning rate.

It is essential to note that when conducting all scenarios, user-inputted lags were incorporated into the model for activities with higher productivity compared to their predecessors, aimed at preventing work stoppages for the successor when it outpaces its predecessor. It is crucial to understand that without these lags, the model would achieve significantly lower project durations. However, this would result in frequent work stoppages whenever the successor outpaces the predecessor. For instance, if Scenario #2 were computed without user-input lags, the project would be completed in only 9,074 h, compared to 15,199 h with the lags. However, the consequence would be frequent work stoppages, as activities G and J would outpace their predecessors, leading to a halt before starting each unit. Figure 8 shows a model run without user-input lags, revealing that activity G experiences idle periods before starting any unit because it outpaces its predecessor. These frequent work stoppages are undesirable in managing projects with repetitive activities, making user-input lags essential. Although these idle periods can impact project duration, they can be beneficial if minimizing the overall project time is the priority, as they allow crew utilization without affecting the main schedule. To determine appropriate lags, various methods can be used. One approach is to run the model, note the predecessor’s last unit finish date, and set this as the successor’s starting date. Alternatively, a parameter can be set to monitor idle time, with multiple model iterations adjusting lags for higher productivity activities to minimize idle time. Both methods yield similar outcomes if all variables remain constant. After simulating the entire project in the same manner, the model showed that completing the project with 4 crews took 16,965 h. This duration is longer than the one calculated in scenario #2, due to the learning loss each time the crew switches activities. Figure 9 illustrates the LOB (Line of Balance) for this scenario as extracted from the model, and Fig. 10 shows the activity duration for each unit for each crew. The activity duration graphs indicate changes when crews are switched, resulting in a decrease in the gained learning momentum. The number of crews used in this scenario was chosen by the user and can be optimized to enhance project performance.

Table 5 compares three scenarios’ results, highlighting how different configurations of learning rates, crew routing, and crew count impact total project duration. In Scenario #1 (Base Case), a traditional Line of Balance (LOB) calculation method is used without incorporating learning effects or crew routing. This approach requires 11 crews and results in a total project duration of 21,800 h, reflecting a straightforward but less efficient scheduling method. Scenario #2 (Incorporating Learning Rate) utilizes AnyLogic simulation with an added learning rate but no crew routing, maintaining the same 11 crews as in the base case. Here, the learning rate helps crews work more efficiently as they repeat tasks, reducing the total project duration to 15,199 h, a significant improvement over Scenario #1. Scenario #3 (Incorporating Crew Routing and Learning Rate) also uses AnyLogic simulation but incorporates both a learning rate and crew routing, which optimizes crew assignments across tasks. This flexibility reduces the required crews to only 4, achieving a project duration of 16,965 h. While slightly longer than Scenario #2, Scenario #3 demonstrates the value of strategic crew routing by achieving substantial resource savings with fewer crews. Overall, the table shows that incorporating learning rates and crew routing can optimize project efficiency, balancing project duration and resource requirements for cost-effective scheduling.

**Table 5:** Scenario 1, 2 and 3 results summaries.

Scenario	Parameters	No. of crews	Total Project Duration /hrs.
Scenario #1 (Base Case)	- Typical LOB Calculation Method - No Learning - No Crew Routing	11	21,800
Scenario #2 (Incorporating Learning Rate)	- AnyLogic Simulation - With Learning - No Crew Routing	11	15,199
Scenario #3 (Incorporating Crew Routing and Learning Rate)	- AnyLogic Simulation - With Learning - With Crew Routing	4	16,965

## Sensitivity analysis

In this section, a sensitivity analysis will be performed to assess the impact of specific parameters on the project’s objective outcome, namely the total project duration. The parameters being evaluated include the Learning Rate Index, Forgetting Rate Index, and the number of crews. Conducting sensitivity analysis is crucial as it helps to understand how changes in key factors can influence project performance, providing valuable insights for decision-making.

### Learning Rate Index sensitivity analysis

**Fig. 11:**
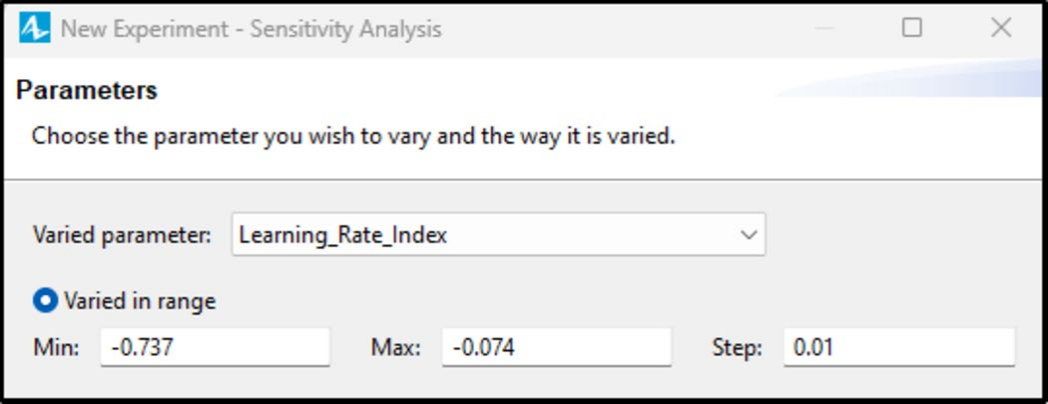
Learning Rate Index sensitivity analysis window.

The analysis will be carried out using the software’s integrated sensitivity analysis window, where the user will input the minimum and maximum values for each parameter, along with the step size. The software will then explore all possible solutions within this range, as demonstrated in Fig. 11.

The values shown in Fig. 10 represent the corresponding Learning Rate Index for a learning rate ranging from 95%, with an index of -0.074, to 60%, with an index of -0.737.

**Fig. 12:**
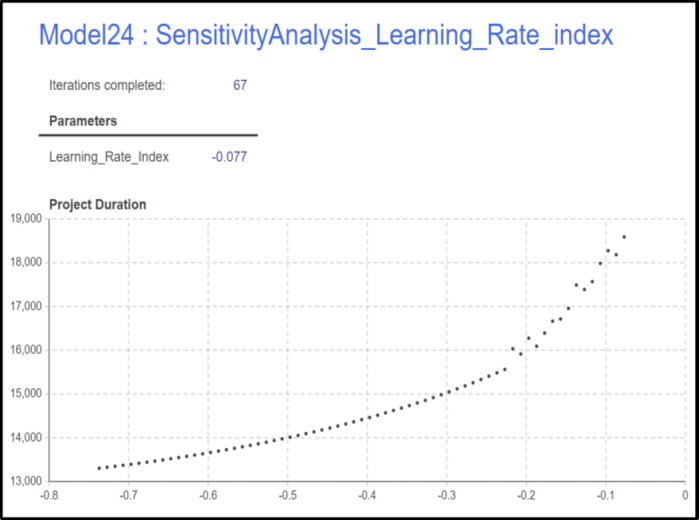
Learning Rate Index sensitivity analysis results.

As anticipated, the project duration is significantly impacted by changes in the Learning Rate Index, as it influences the exponential reduction in activity duration resulting from the learning effect. The values from this experiment, displayed in Fig. 12. The figure illustrates how the total project duration decreases as the Learning Rate Index changes. A total of 67 iterations were completed, representing the possible scenarios between the minimum Learning Rate Index of -0.737 and the maximum of -0.074, with a step of 0.01. This results in 67 total iterations.

### Forgeting rate sensitivity analysis

The analysis will be conducted using the software also with the sensitivity analysis tool, where the user specifies the minimum and maximum values for each parameter, as well as the step size. The software will then generate all possible solutions within this defined range, the range in use is 0.074 as the min value and 0.737 and 0.01 step.

**Fig. 13:**
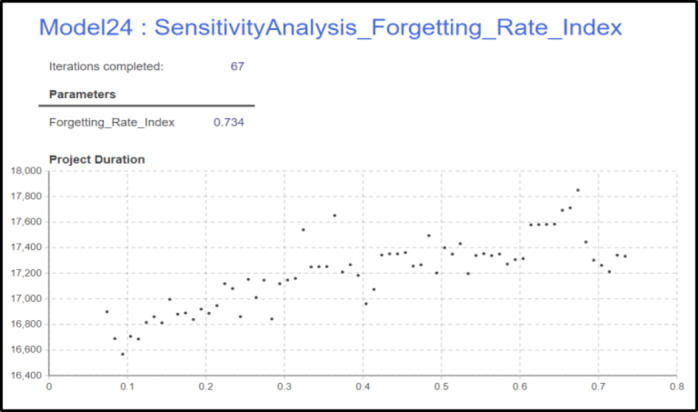
Forgetting Rate Index sensitivity analysis results.

The sensitivity analysis shown in Fig. 13, demonstrates the relationship between the Forgetting Rate Index and project duration. As the Forgetting Rate Index increases, there is a noticeable upward trend in project duration. This suggests that as the forgetting effect becomes more pronounced (higher index), crews lose efficiency more rapidly, which extends the time required to complete the project. Notably, the project duration varies between approximately 16,400 and 17,800 h across the range of Forgetting Rate Index values. This indicates that higher forgetting rates significantly impact crew productivity, potentially due to the reduced retention of skills or process familiarity when crews are rerouted across activities. The scatter of points at higher values of the Forgetting Rate Index shows more variability in duration, suggesting that at higher forgetting rates, other factors (such as the nature of the crew routing) may further influence performance. This analysis underscores the importance of managing and minimizing the forgetting effect in projects involving repetitive activities and crew routing. Lower Forgetting Rate Indices appear to contribute to shorter project durations, highlighting the value of strategies that can maintain crew efficiency over time, such as regular training or consistent task assignments.

### Crews sensitivity analysis

Conducting a sensitivity analysis on the number of crews in use is crucial for optimizing project performance in repetitive construction projects with varying crew types. By evaluating how changes in crew numbers impact project duration, this analysis allows us to make informed decisions on resource allocation and crew deployment strategies. Given that we have different crew types, each with specific skills and efficiencies suited to various tasks, adjusting the number of crews per type can significantly influence productivity, project timelines, and cost-efficiency. Assuming a minimum of one crew and a maximum of ten crews for each type, we plan to conduct four separate sensitivity analyses, one for each crew type. This approach will allow us to observe how changes in crew size for each specific type impact project duration and whether adding more crews results in diminishing returns or continues to reduce the project timeline effectively. The results of this analysis will also enable us to determine the optimal number of crews per type, balancing the benefits of shorter project duration against the cost implications of hiring additional crews. In cases where adding crews results in minimal time savings or incurs high additional costs, we might consider reallocating resources to other crew types or exploring alternative strategies, such as staggered crew shifts. Ultimately, this sensitivity analysis on crew numbers provides insights into the relationship between crew allocation and project duration, helping ensure that we deploy an effective and efficient workforce. It enables us to better manage crew routing and maximize productivity, contributing to a well-balanced and cost-effective project execution. Table 6 shows the crew numbers and corresponding project duration for each type.

**Table 6:** Crew number sensitivity analysis results.

No of Crews	Crew Type 1	Crew Type 2	Crew Type 3	Crew Type 4
1	16852.22	16852.22	16852.22	16852.22
2	16228.44	16810.02	16228.44	16371.6
3	16228.44	16810.02	16228.44	16371.6
4	16228.44	16810.02	16228.44	16371.6
5	16228.44	16810.02	16228.44	16371.6
6	16228.44	16810.02	16228.44	16371.6
7	16228.44	16810.02	16228.44	16371.6
8	16228.44	16810.02	16228.44	16371.6
9	16228.44	16810.02	16228.44	16371.6
10	16228.44	16810.02	16228.44	16371.6

Table 6 summarizes the impact of increasing the number of crews for each crew type on the project duration. For example, the project duration decreases significantly when the number of crews increases from 1 to 2, dropping from 16,852.22 h to 16,228.44 h. However, beyond two crews, there is no further reduction in project duration, indicating that adding more crews of this type does not enhance productivity after reaching two crews in crew Type 2, similar trend is observed for Crew Type 2, where increasing from 1 to 2 crews reduces the project duration from 16,852.22 h to 16,810.02 h. Further increases in the number of crews for this type do not impact the project duration, suggesting a saturation point at two crews for this crew type. Same findings with crew 3 and crew 4. The results indicate that adding more than two crews per type does not lead to additional reductions in project duration, suggesting an optimal crew size of two for each crew type. This outcome highlights a diminishing returns effect, where increasing the number of crews beyond two does not enhance productivity or efficiency. Therefore, to optimize resource allocation and minimize costs, it is recommended to limit the number of crews to two for each type in this project.

However, this assumption may not hold true when conducting multiple trials with simultaneous changes in crew numbers across all types. Adjusting the number of crews for all types concurrently could lead to a different project duration. The previous results occurred because increasing the number of one type of crew without proportionately increasing the others does not yield significant improvements. Therefore, a comprehensive sensitivity analysis involving simultaneous adjustments across all crew types is essential to uncover potential efficiencies that may arise from a more balanced increase in crew deployment.

## Model verification and validation

This section is organized into two key subsections: model verification and model validation. In the model verification subsection, several iterations will be performed to ensure that the model functions as intended, confirming its accuracy and preventing any unexpected or anomalous outcomes. The model validation subsection will focus on testing the model against real-world data, assessing how well the model’s results align with actual outcomes to ensure its reliability and accuracy in practical applications.

### Model verification

In this section, model verification will be carried out in two steps. The first step involves calculating the project duration using the base case assumption of assigning one crew per crew type. In the second step, Base Case as shown in Table 7, will be performed with a total number of crews greater than in the base case. This trial is expected to result in a shorter project duration compared to the duration calculated in the base case module. The second step involves comparing iteration 2 with three other iterations, all of which have the same total number of crews but with different crew type configurations Table 7. This comparison should yield different results for each scenario. These trials, based on user input, can be applied to the model to perform a sensitivity analysis, assessing the impact of increasing each crew’s capacity (number of available crews). This serves as a preliminary step before conducting a full model optimization to achieve the optimal results as shown in Table 8, the base case, where 4 crews were assigned (1 per crew type), resulted in a total project duration of 16,965 h. When compared to the second trial (Iteration 1), which used a total of 7 crews, there was a significant reduction in project duration, bringing it down to 9,432 h. However, the other three iterations, which also used 7 crews but with different crew configurations, produced different results from Iteration. This demonstrates that the total number of crews is not the sole factor controlling the project duration as calculated by the model. This outcome was anticipated to ensure the model was functioning as expected. Therefore, in the optimization phase, it was crucial not only to determine the total number of crews but also to establish the optimal configuration and crew movement. This confirms that the data from the optimization module led to the best possible project duration.

**Table 7:** Model verification trials set.

	Crew 1 (Capacity)	Crew 2 (Capacity)	Crew 3 (Capacity)	Crew 4 (Capacity)
Base Case	1	1	1	1
Iteration 1	2	1	2	2
Iteration 2	3	1	1	2
Iteration 3	4	1	1	1
Iteration 4	1	2	1	3

**Table 8:** Model verification trials results.

	Project Duration
Base Case	16,965
Iteration 1	9,432
Iteration 2	11,659
Iteration 3	12,058
Iteration 4	14,177

### Model validation

This section is crucial for ensuring the accuracy and reliability of the developed model. By testing the model against a real case study, it provides an opportunity to verify how well the model’s predictions align with actual project outcomes. This step is essential for establishing confidence in the model’s ability to perform in real-world scenarios, ensuring that its results are not only theoretically sound but also practical and applicable to real-life projects. Successful validation strengthens the credibility of the model and confirms its usefulness for decision-making in similar future projects.

In their study, K.C. Lam, Donald Lee, and Tiesong Hu explore the impact of learning and forgetting phenomena on productivity in repetitive construction operations^13^. They argue that the conventional assumption of consistent unit production times, both before and after an interruption, neglects the effects of these phenomena. By integrating the learning-forgetting model using a line of balance technique, they provide a method to predict productivity losses and offer an alternative approach for managing works, resources, and cash flow in construction projects. They presented a table detailing the start and end dates for each unit in their 30-unit project. They assumed a 30-day work stoppage following the completion of the 20th unit. The start and end dates, along with activity durations, the parameters used in the research include a forgetting coefficient of 0.15, a 90% learning rate, and 15 days required to produce the first unit. These are summarized in Table 9. To validate the accuracy of our paper’s model, the same case study will be solved using our methodology.

**Table 9:** Parameters coefficients used in the model.

Parameter	Coefficient
Forgetting coefficient parameter	0.15
Learning rate	90%
Time to produce first unit	15 days

The same parameters will be applied in our model to provide a clear comparison between the two methodologies and assess how the model behaves when a stoppage occurs after unit 20. As shown in previous work, the unit duration decreased from 15 days to 8.1 days by unit 20. Due to the forgetting effect after the work stoppage, the duration increased from 8.1 days to 14.58 days, then gradually decreased again, reaching 8.78 days by unit 30.

As noted in Table 10, the data extracted from the AnyLogic model closely aligns with the data presented in previous work. The model achieved an activity duration of 7.996 days for the first 20 units without work stoppages, compared to 8.1 days in the previous study. After the work stoppage, the model showed a loss of learning due to forgetting, with the activity duration increasing to 14.65 days, compared to 14.58 days in the earlier work. Learning then accumulated again, bringing the duration down to 9.033 days, in comparison to 8.78 days in the previous study. Several factors influence the accuracy and similarity of the results. One key factor is the model used for calculating the learning rate, along with the forgetting model applied. The model solved in the previous research^13^, the total activity duration for the total 30 units with the work stoppage is 323 days, and in this research the duration is 325 days. Additionally, incorporating more complex learning and forgetting models with additional parameters could improve the results and provide greater precision. The Line of Balance (LOB) extracted from the AnyLogic model for the case study mentioned above, shown in Fig. 10, illustrates the activity durations for each unit and highlights the impact of both learning and forgetting effects.

## Research contribution

Considering previous work in this area, this research offers a more robust contribution by leveraging simulation software to address more complex problems than those tackled in earlier studies. For example, the research by^13^ focused on a project with only three activities to keep the solution simple, as the manual equations used would be challenging to apply to larger, more complex projects. Similarly, in Gouda’s research^3^, when multiple crews were used, implementing the effects of learning and forgetting rates was difficult due to the limitations of the proposed methodology. Additionally, this research provides a strong visual representation of the solution through simulation software, which can be further enhanced according to user needs, offering a more flexible and intuitive approach to problem-solving.

## Research limitations and recommendations

This research is limited by several factors. Firstly, the learning and forgetting models used are basic, with the Wright model for the learning rate and the LFL model for the forgetting rate. These models were chosen for simplicity and to demonstrate the theory. More complex models could be implemented for greater accuracy and realism. For instance, some forgetting models account for additional factors such as labor skill, task complexity, and environmental conditions, which are critical for obtaining more realistic values. Another limitation is the absence of optimization techniques to compare the results and assess the accuracy and advantages of the extracted model. Finally, the model is not fully generic, as it requires building a new project setup for each specific case, with all data extracted manually. A more practical approach would be to develop a user-friendly interface to streamline the optimization process. The AnyLogic working environment enables seamless management of data visualization and calculation methods.

**Table 10:** Start, end and durations of each activity after integrating learning and forgetting [15] and model results.

unit no.	Ideal Case			Forgetting and learning			AnyLogic Model Durations
	Start	Finish	Duration	Start	Finish	Duration	
1	0	9.1	9.1	0	15	15	15
2	9.1	18.2	9.1	15	27	12	12.968
3	18.2	27.3	9.1	27	38.08	11.08	11.91
4	27.3	36.4	9.1	38.08	48.6	10.52	11.211
5	36.4	45.5	9.1	48.6	58.72	10.12	10.698
6	45.5	54.6	9.1	58.72	68.54	9.82	10.296
7	54.6	63.7	9.1	68.54	78.11	9.57	9.968
8	63.7	72.8	9.1	78.11	87.48	9.37	9.693
9	72.8	81.9	9.1	87.48	96.67	9.19	9.456
10	81.9	91	9.1	96.67	105.7	9.03	9.249
11	91	100.1	9.1	105.7	114.6	8.9	9.066
12	100.1	109.2	9.1	114.6	123.38	8.78	8.902
13	109.2	118.3	9.1	123.38	132.04	8.66	8.753
14	118.3	127.4	9.1	132.04	140.61	8.57	8.618
15	127.4	136.5	9.1	140.61	149.08	8.47	8.494
16	136.5	145.6	9.1	149.08	157.46	8.38	8.38
17	145.6	154.7	9.1	157.46	165.77	8.31	8.274
18	154.7	163.8	9.1	165.77	174	8.23	8.175
19	163.8	172.9	9.1	174	182.17	8.17	8.083
20	172.9	182	9.1	182.17	190.27	8.1	7.996
*30 days work stoppage*							
21	212	221.1	9.1	220.27	234.85	14.58	14.65
22	221.1	230.2	9.1	234.85	246.51	11.66	12.665
23	230.2	239.3	9.1	246.51	257.28	10.77	11.632
24	239.3	248.4	9.1	257.28	267.5	10.22	10.95
25	248.4	257.5	9.1	267.5	277.35	9.85	10.448
26	257.5	266.6	9.1	277.35	286.89	9.54	10.056
27	266.6	275.7	9.1	286.89	296.19	9.3	9.736
28	275.7	284.8	9.1	296.19	305.3	9.11	9.466
29	284.8	293.9	9.1	305.3	314.23	8.93	9.235
30	293.9	303	9.1	314.23	323.01	8.78	9.033
Total Duration						**323.01**	**325.87**

## Conclusions

This study extensively investigates the application and optimization of the Line of Balance (LOB) technique for scheduling and managing repetitive projects. Through a series of methodical trials, the study highlights the significant impacts of learning rates, forgetting rates, and crew routing on project duration and resource efficiency. The initial scenario, using traditional LOB methods without accounting for the learning effect or allowing for crew routing, resulted in a baseline project duration of 21,800 h. The second scenario incorporated an 80% learning rate, resulting in a substantial reduction in project duration to 15,218 h. This scenario underscored the importance of maintaining uninterrupted workflow, demonstrating how the learning effect enhances productivity and reduces activity durations over time. The third scenario introduced crew routing among activities, aimed at reducing the number of crews required and enable crews to transition between activities upon completion, allowing them to move to a different task to minimize the number of crews needed, Hence, minimizing the indirect costs associated with hiring and firing crews. Despite these economic benefits, this approach led to a longer project duration of 16,965 h compared to the second scenario. The extended duration was due to disruptions in learning momentum each time crews switched tasks, highlighting the trade-off between number of crews needed and project duration (Fig. 5).

In summary, while the LOB technique excels in maintaining a consistent workflow and reducing project duration through the learning effect resulted from maintaining work continuity and preventing work stoppages or crew routing, optimizing crew routing could result in a better project resources utilization. However, this optimization requires careful consideration. Decision-makers must balance the trade-offs between preserving learning rate momentum with dedicated crews and the economic advantages of crew routing. By meticulously evaluating factors such as learning and forgetting rates, indirect costs, and project deadlines, project managers can make informed decisions to achieve the optimal balance between project duration and cost-effectiveness. This approach ensures the best possible project outcomes, aligning with both time and budget constraints to further refine these findings, In this study, the researcher experimented with various crew numbers to minimize the required crews in scenario #2 while aiming to preserve the learning momentum and project duration attained in scenario #2. future work will include multiple trials with increasing crew numbers to explore their effects on project outcomes more comprehensively.

In summary, the trials conducted for this case study demonstrated that adhering to the traditional LOB calculation method and avoiding any crew routing that disrupts learning momentum yields the shortest project duration. However, this approach requires the maximum number of crews, as it necessitates a dedicated crew for each activity. On the other hand, allowing crew routing between activities can significantly reduce the number of crews needed, but it results in a longer project duration due to the loss of learning momentum when crews transition between different tasks. Despite this, the reduction in crew numbers can lower project costs and enhance resource utilization. A balanced approach can be achieved by exploring and comparing various scenarios with changing the crew numbers and try all the different routing maps possible. The use of simulation software proved beneficial, as the results obtained could not be easily calculated using typical methods such as Excel or MATLAB. This is due to the complexity of the equations involved in calculating learning and forgetting rates, and the difficulty in recalculating these equations every time there is a work stoppage during the project lifespan while maintaining the relationships between activities. The research does have some limitations. The model used is not fully generic and must be rebuilt for each individual project. Additionally, alternative methods for calculating the forgetting rate may offer more accurate results, as they consider a broader range of factors influencing the duration of learning loss. Furthermore, the model could be improved to better track crew movements between activities, as extracting this data from the current model is complex and requires manual intervention.

## Data Availability

The data that support the findings of this study are openly available in Zenodo at https://doi.org/10.5281/zenodo.13335681.
